# Clinical comparison of adaptive 4DCBCT scanning protocols for lung tumor motion assessment

**DOI:** 10.1002/acm2.70172

**Published:** 2025-07-14

**Authors:** Sadia Sana, Owen Dillon, Ricky T. O'Brien

**Affiliations:** ^1^ School of Health and Biomedical Sciences RMIT University Melbourne Australia; ^2^ School of Health Sciences University of Sydney New South Wales Australia

**Keywords:** adaptive 4DCBCT, lung tumor motion, image‐guided radiotherapy, motion‐compensated reconstruction, respiratory motion tracking

## Abstract

**Background:**

Adaptive four‐dimensional cone beam computed tomography (4DCBCT) has been proposed as a novel method to reduce imaging dose and scan time. This technology involves both adaptive imaging and motion‐compensated reconstruction. However, no study has been performed in a lung cancer patient cohort to confirm that adaptive 4DCBCT accurately images tumor motion. This is partly due to a lack of a ground truth comparison and the difficulty of assessing 4DCBCT images. This issue will be addressed in this study.

**Purpose:**

This study aims to investigate and assess lung tumor motion in adaptive four‐dimensional cone beam computed tomography (4DCBCT) across two treatment days using data from the ADAPT clinical trial. Tumor motion measured in reconstructed adaptive 4DCBCT images was compared to motion observed in conventional 4‐min 4DCBCT scans to evaluate the accuracy and feasibility of adaptive imaging for clinical use.

**Methods:**

Lung tumor motion from the ADAPT clinical trial was compared between conventional 4DCBCT (1320 projections in 4 min) and adaptive 4DCBCT for different adaptive scan types [a fast 200 projection adaptive 4DCBCT scan (ADAPT_200) acquired in approximately 60 s and a slower, 600 projections adaptive 4DCBCT scan acquired in approximately 4 min (ADAPT_600)]. The fast 200 projection adaptive 4DCBCT was reconstructed using a motion‐compensated image reconstruction algorithm, while the conventional and slower 600 projection scans were reconstructed with the FDK algorithm. Analysis was performed across two fractions (f1 and f2) on different days. For each scan type, the center of mass (COM) trajectory was computed for each respiratory phase to quantify tumor motion. The mean and standard deviation difference in tumor COM between the conventional and adaptive 4DCBCT scans were used to quantify tumor trajectory accuracy.

**Results:**

For the fast adapt scan (ADAPT_200), 80.4% of fractions showed motion differences of less than 1 mm from the conventional scan, 13.3% had motion differences of 1–2 mm, and 6.7% had motion differences greater than 2 mm. For the high‐quality adapt scan (ADAPT_600), 76.9% of fractions showed motion differences of less than 1 mm from the conventional scan, 15.4% had motion differences of 1–2 mm, and 7.7% had motion differences greater than 2 mm.

**Conclusion:**

Adaptive 4DCBCT with as low as 200 projections can image the tumor's trajectory, however, minor deviations were observed, with 13.3% of cases showing motion differences of 1–2 mm and 6.7% exceeding 2 mm. These findings demonstrate that faster acquisition (60 s) of adaptive 4DCBCT is feasible for a majority of lung cancer radiation therapy patients.

## 1 INTRODUCTION

Four‐dimensional cone beam computed tomography (4DCBCT) has proven particularly useful for patient positioning and image guidance in lung cancer radiotherapy^1^ While 4DCBCT is effective in guiding lung cancer radiotherapy treatments,^2^, ^3^ it suffers from poor image quality,^4^ long scan times,^5^ and high imaging doses.^4^, ^6^, ^7^

Recent investigations indicate promising outcomes with adaptive 4DCBCT,^8^, ^9^, ^10^ which adapts the image acquisition hardware to real‐time patient breathing rate changes. For example, if the patient breathes faster, the gantry rotation and projection acquisition are sped up. By incorporating motion‐compensated reconstruction methods^11^ alongside adaptive imaging approaches,^12^ substantial reductions in imaging dose by up to 85% and scan duration by up to 75% are achievable, while concurrently maintaining or even enhancing image quality compared to prevailing clinical standards.^8^, ^13^

A key clinical use of 4DCBCT for lung cancer radiation therapy is to see where the tumor moves. Although adaptive 4DCBCT has been studied in terms of contrast‐to‐noise and edge sharpness,^14^, ^15^ there has not been a systematic study to determine if adaptive 4DCBCT represents tumor motion accurately. This study aims to investigate and assess tumor motion in adaptive 4DCBCT across two days of treatment from the ADAPT clinical trial. The centre of mass (COM) has been used as a way to measure the tumor location, with the x, y, and z values of the COM representing the tumor location. We found this approach more reliable than automated segmentation tools, especially when there is nearby soft tissue. Additionally, intra‐fraction motion variations, including baseline shifts and respiratory irregularities, were analyzed to ensure a comprehensive evaluation. By incorporating patient‐specific motion patterns into the analysis, this study provides valuable insights into the effectiveness of adaptive 4DCBCT in capturing tumor motion. A better understanding of its accuracy can enhance treatment planning and optimize radiation delivery, ultimately improving patient outcomes.

## 2 METHODOLOGY

**FIGURE 1:**
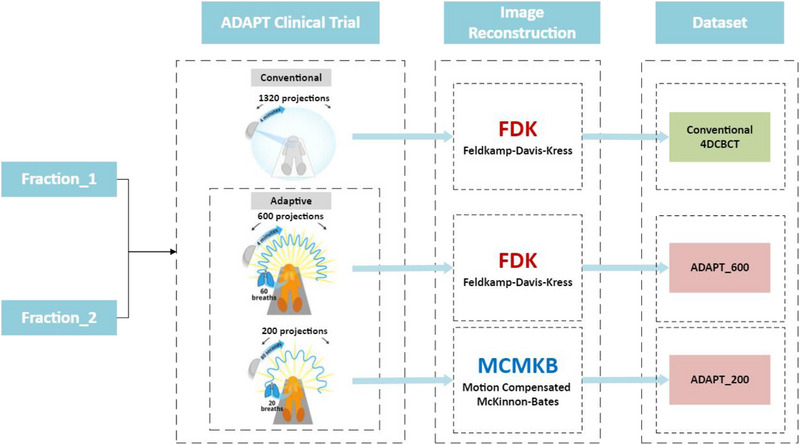
The 4DCBCT Datasets: Lung cancer radiation therapy patients receive a conventional 4DCBCT scan for patient positioning, and the 200‐projection and 600‐projection adaptive 4DCBCT acquisitions are acquired immediately after the conventional 4DCBCT.

Figure 1 is a schematic of how the 4DCBCT datasets were acquired and reconstructed for the ADAPT clinical trial.

### 2.1 The ADAPT clinical trial

The ADAPT clinical trial acquired a conventional 4‐minute 4DCBCT scan for the patient's first day of treatment, conventional 4DCBCT scan was performed as part of the standard patient treatment. Additionally, on the same day, two adaptive scans were acquired: (1) a fast, 200‐projection adaptive scan and (2) a high‐quality, 600 projection adaptive scan. A second round of scans, including one conventional 4DCBCT scan and two adaptive scans, was performed on a later treatment day. In total, five additional scans were acquired across two treatment days across 30 lung cancer patients treated at Liverpool Hospital (Australia). The conventional 4DCBCT scan has an effective dose of 100%. The ADAPT_200 fast scan delivers 15%–30% of the conventional dose, while the ADAPT_600 high‐quality scan delivers approximately 50%.

The ADAPT_600 scan was selected to represent an adaptive acquisition with a similar scan time to the conventional scan (approximately 4 min) with less radiation dose. This acquisition provides the opportunity for clinics to halve the imaging dose within a similar patient scan time as current clinical practice.

### 2.2 Conventional 4DCBCT acquisition (Conventional 4DCBCT)

In accordance with Liverpool Hospital's established patient treatment protocol, conventional 4DCBCT scans were obtained. The conventional 4DCBCT scans involved 1320‐projection over a 200° arc during a 4‐min rotation. The conventional projections were subsequently retrospectively sorted utilizing Elekta's x‐ray volume imaging software (XVI), employing the Amsterdam Shroud method^16^ and offline reconstruction was performed using the FDK algorithm in the Reconstruction Toolkit.^17^, ^18^ No postprocessing of the reconstructed image is performed to ensure that we are comparing only the acquisition and reconstruction approaches.

### 2.3 The high‐quality adaptive 4DCBCT acquisition (ADAPT_600)

Following treatment, patients underwent a 600‐projection adaptive 4DCBCT scan where the gantry rotation speed was optimized and modulated to real‐time changes in the patient's breathing rate. This method, described in the first patient treatment study^19^ allowed for adaptive acquisition in approximately 4 min, similar to the conventional 4DCBCT scan duration. ADAPT_600 acquired 60 projections in 10 respiratory phases across 60 breathing cycles (approximately 4 min). The offline reconstruction was performed using the FDK algorithm in the Reconstruction Toolkit.^17^, ^18^ No postprocessing of the reconstructed image is performed to ensure that we are comparing only the acquisition and reconstruction approaches. ADAPT_600 acquires 45% of the projections of the conventional 4DCBCT scan in approximately the same amount of time (4 min).

### 2.4 The fast adaptive 4DCBCT acquisition (ADAPT_200)

The ADAPT_200 scan utilized an optimized fast adaptive 4DCBCT method to reduce scan time while maintaining image quality. In this technique, 20 projections were captured for each of the 10 respiratory phases, resulting in 200 projections over a 200‐degree gantry rotation arc. ADAPT_200 had a scan duration of approximately 60–80 s across 20 patient breathing cycles. Compared to the conventional 1320‐projection acquisition, ADAPT_200 reduced the projection count by 85%. Motion‐compensated image reconstruction was performed based on the methodology described in previous studies.^19^, ^20^ We have used MCMKB as a modern reconstruction technique that can reconstruct images with a low projection count. In simulation studies, we found that MCMKB was able to acquire projections with as low as 200 projections (85% fewer than conventional acquisition). We selected ADAPT_200 with MCMKB to test image acquisition with the lowest projection count possible.

The ADAPT images are prospectively sorted with the projection acquisition controlled and triggered to acquire each projection as close to the center of each respiratory bin as possible.

### 2.5 Measuring tumor motion

**FIGURE 2:**
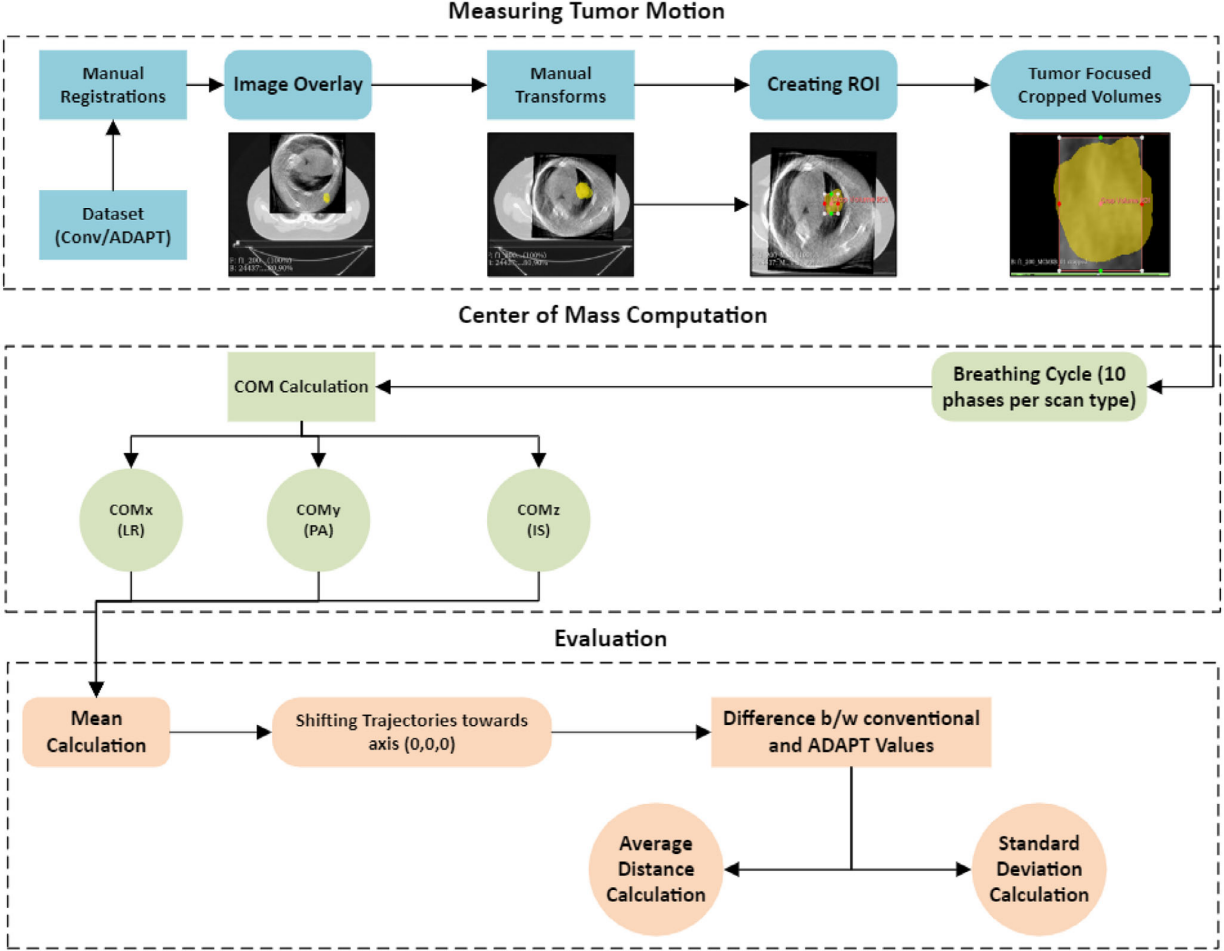
Flowchart shows the tumor motion analysis and comparison approach. The process involves measuring tumor motion through manual registrations, image overlays, and creating regions of interest (ROI). The center of mass (COM) was calculated for each phase of the breathing cycle, and the motion data was evaluated to compare conventional and adaptive imaging methods, focusing on average distance and standard deviation.

Figure 2 is a schematic of the method used to measure the tumor motion in the 4DCBCT scans, which we will describe in detail below. Data analysis was conducted using MATLAB and 3D Slicer (5.2.2) software tools for registering 4DCBCT and 4DCT images. As detailed below, the process involved several key steps, including rigid registration, region of interest (ROI) selection, tumor cropping, center of mass (COM) calculation, and motion comparison between conventional and ADAPT scans.

#### 2.5.1 Rigid registration

The initial step involved aligning the 4DCT to the 4DCBCT image using the rigid registration tools in 3D Slicer (5.2.2).

The registration was performed using the “General Registration (BRAINS)” module in 3D Slicer.

The 4DCT image was designated as the fixed image, while the 4DCBCT image was set as the moving image. Rigid registration was then initialized using the default parameters to achieve an initial alignment before further manual adjustments were made by:

1. Overlaying the 4DCBCT image onto the 4DCT image.
2. Systematic manual adjustments in AP, SI, and LR were made using the “Transforms” module.
3. Small rotational adjustments around each axis were then performed to better align anatomical structures.
4. Each adjustment was visually verified in multiple planes (axial, coronal, and sagittal) to ensure that the anatomical features, particularly the tumor, were accurately aligned.

#### 2.5.2 Creating regions of interest (ROIs) and cropping the tumor

With the images accurately registered, the next step involved creating regions of interest (ROIs) around the tumor and cropping the images accordingly.

1. Using the “Segment Editor” module in 3D Slicer, ROIs were manually delineated around the tumor in both the 4DCT and registered 4DCBCT images.
2. The defined ROIs were then used to crop the images, isolating the tumor area from surrounding tissues and bony anatomy. This was done using the “Crop Volume” module, focusing the analysis on the tumor region. The image was initially cropped around the ITV to encapsulate it within the smallest possible cube containing the ITV. Size adjustments of the ROI were then made to eliminate bony anatomy if necessary. During this process, if the size adjustments failed to remove bony anatomy from the internal target volume (ITV), the images were rejected. This was determined through a visual inspection of all three planes. If any bony structures remained within the cropped region intended to isolate the tumor, those images were considered unsuitable for further analysis and excluded from the study. Bony anatomy in the ROI can interfere with accurate tumor delineation due to its high density and distinct imaging characteristics, which may lead to artifacts and inaccuracies in the motion estimation and registration processes. Thus, to ensure precise measurement of tumor motion and avoid potential errors, it is crucial to exclude any images where bony structures are present in the cropped region. This stringent verification ensured that only images with clear and accurate tumor representation, free from bony anatomy, were included in the final dataset for further analysis.
3. The cropped images were visually reviewed to ensure that the ROIs were accurately defined and that the resulting images were suitable for further analysis.

#### 2.5.3 Centre of mass computation

The COM for each of the 10 respiratory phases was calculated in three‐dimensional space. The following steps were taken to determine the COM coordinates along the LR, PA, and IS axes.

The following steps were taken to determine the COM coordinates along the LR, PA, and IS axes. A MATLAB script was used to compute the COM of the tumor across each phase. The approach sums the voxel intensities along each dimension to find the weighted positions using:

$$\begin{array}{cc} \mathrm{COMx} & =\frac{\sum_{i}\mathrm{xi}\cdot \mathrm{Ii}}{\sum_{i}\mathrm{Ii}},\;\mathrm{COMy}=\frac{\sum_{i}\mathrm{yi}\cdot \mathrm{Ii}}{\sum_{i}\mathrm{Ii}},\\ \mathrm{COMz} & =\frac{\sum_{i}\mathrm{zi}\cdot \mathrm{Ii}}{\sum_{i}\mathrm{Ii}} \end{array}$$

Here, ***x_i_***, ***y_i_***, and ***z_i_*** ​ represent the coordinates of voxel ***i***, and ***I_i_*** represents the intensity of voxel 𝑖.

The COM coordinates (COMx, COMy, COMz) for each phase and scan type were analyzed to determine the variability and consistency across different fractions and imaging techniques. Here, COMx, COMy, and COMz represent the COM values for the planes LR, PA, and IS, respectively.

The COM was computed based on the segmented tumor ROI to minimize the influence of non‐tumor tissues. This approach ensured that motion estimation was focused on the tumor itself rather than surrounding structures. However, as noted in some cases, minor contributions from adjacent lungs or soft tissue may have affected the COM calculation. This limitation highlights the challenge of accurately isolating tumor motion, particularly in cases with heterogeneous voxel intensity distributions. Interestingly, in most patients where the tumor exhibited clear boundaries and could be cropped without including surrounding tissues, we observed a consistent motion pattern across different scan types. This consistency reinforces the reliability of adaptive 4DCBCT in capturing tumor motion, particularly in well‐defined cases.

### 2.6 Calculating the difference between tumor motion in the conventional and ADAPT scans

The COM coordinates (COMx, COMy, COMz) for each phase and each scan type were determined as described above. As the conventional scan was acquired before a couch shift, and the patient may have moved before the adaptive acquisitions, we subtract the mean COM position (to account for the variation in the anatomy captured within the ROI and the contrast between scans) from each scan to get a more representative tumor motion range for each scan. This was achieved by calculating the mean COM position from each scan and then subtracting the mean position from the COM for each respiratory phase:

Normalized COMx = COMx − mean_COMx
Normalized COMy = COMy − mean_COMy
Normalized COMz = COMz − mean_COMz

Finally, the difference in tumor motion between the conventional and adapt scans was computed for each phase using the Euclidean distance: $d=\surd [{\left(\mathrm{COMx}\_\mathrm{conv}-\mathrm{COMx}\_\mathrm{adapt}\right)}^{2}+{\left(\mathrm{COM}y_{\mathrm{conv}}-\mathrm{COM}y_{\mathrm{adapt}}\right)}^{2}+{\left(\mathrm{COM}z_{\mathrm{conv}}-\mathrm{COM}z_{\mathrm{adapt}}\right)}^{2}]$

The distances were calculated for each phase across all patients, and then the average distance for each phase was computed. This provided an overall measure of the difference between the conventional and adaptive methods:

$$\mathrm{Average}\;\mathrm{Distance}=\frac{1}{N}\;\sum_{i=1}^{N}\mathrm{di}$$

where *N* is the number of phases (10 in this study), and 𝑑𝑖 is the distance for each phase.

The standard deviation 𝜎 was calculated to quantify the dispersion of distances around the mean, providing information about the variability:

$$\sigma =\surd \frac{1}{N}\;\sum_{i=1}^{N}{\left(\mathrm{di}-\mathrm{Average}\;\mathrm{Distance}\right)}^{2}$$

The inclusion of standard deviation allows for a more comprehensive understanding of the spread of distances and the consistency of the differences between the conventional and adaptive methods across different phases.

## 3 RESULTS

After cropping the patient images, we were left with 16 patients who had no bony anatomy in the cropped region for further analysis. Among these 16 patients, 30 fractions have an ADAPT_200 scan, while 26 fractions, 46.43% have an ADAPT_600 scan for further analysis.

Table 1 summarizes the demographic and clinical characteristics of the 16 patients. The cohort comprised nine males and seven females, with ages ranging from 52 to 81 years at the time of consent. The average age was 69.3 years. Patient body weights varied, with a mean weight of 75.7 kg, ranging from 52.2  to 95.1 kg.

**TABLE 1:** Patient data obtained from the Australia New Zealand Clinical Trial Registry.

					Total motion amplitude (mm) in conventional 4DCBCT	
Patient no.	Age years (till consent date)	Sex	Weight (kg)	Tumor stage	f1	f2
1	71	Male	94.4	T2N2M0	4.45	0.70
3	59	Female	63.8	T3N2M0	0.76	1.17
8	58	Female	95.1	T1N2M1	0.66	0.75
11	52	Female	70.1	T2N1M1	0.00	5.12
13	74	Female	52.2	T1N0M0	1.68	2.29
14	78	Male	93	T1N0M0	0.96	0.98
17	77	Female	59.9	T1N0M0	0.41	0.88
18	78	Male	69	T1N0M0	1.65	1.61
20	63	Male	81.4	T4N2M0	1.91	1.95
21	67	Male	74.6	T2N1M0	0.11	0.57
24	72	Male	62.5	T0N3MX	2.34	2.42
25	77	Female	55	T1N0M0	1.14	1.02
26	81	Female	84	T1N0M0	1.34	1.20
28	74	Female	75	T4N0M1	0.32	0.74
29	59	Male	81	T2N1M0	2.48	0.92
30	70	Male	83.8	TXN2M0	1.69	1.79

The cancer tumor stages were distributed as follows: one patient at stage T0, nine patients at stage T1, four patients at stage T2, 1 patient at stage T3, two patients at stage T4, and one patient at stage TX.

To enable comparison of tumor motion characteristics across imaging protocols, we quantified the total motion amplitude of the tumor COM in conventional 4DCBCT for all 16 patients. The motion amplitude was calculated for two fractions. The mean motion amplitude across patients was with a range from 0.00  to 5.12 mm. Notably, the largest observed motion occurred in patient 11 (f2 = 5.12 mm), while some patients showed minimal motion (e.g., Patient 11 at f1 = 0.00 mm). These values provide a reference for evaluating motion consistency and differences in amplitude when comparing with the ADAPT_200 and ADAPT_600 acquisition strategies, as explored in subsequent sections.

### 3.1 The average difference between tumor trajectories across the different scan types

Table 2 provides a summary of the average distances and standard deviations for each patient, separated by scan type ADAPT_200 and ADAPT_600, and a fraction (f1 and f2). These average distances represent the mean positional differences of the tumor's COM across different phases.

**TABLE 2:** The average differences between the conventional and adaptive scans across the patient cohort.

Patient no.	Fraction	Scan type	Average distance	Std. deviation	Patient no.	Fraction	Scan type	Average distance	Std. deviation
Patient_01	f1	200	2.4	0.6	Patient_21	f1	200	0.5	0.7
		600	2.3	0.6			600	1.2	0.5
	f2	200	0.5	0.4		f2	200	0.3	0.2
		600	0.4	0.3			600	0.2	0.1
Patient_03	f1	200	0.3	0.1	Patient_24	f1	200	2.0	1.3
		600	0.2	0.1			600	1.1	0.2
	f2	200	0.4	0.2		f2	200	1.5	0.5
		600	0.3	0.1			600	1.3	0.3
Patient_08	f1	200	0.7	0.5	Patient_25	f1	200	0.5	0.3
		600	0.8	0.2			600	0.6	0.4
	f2	200	0.2	0.1		f2	200	0.3	0.1
		600	0.1	0.1			600	0.3	0.2
Patient_11	f2	200	1.5	1.5	Patient_26	f1	200	0.6	0.5
Patient_13	f1	200	0.6	0.3			600	0.8	0.6
		600	0.9	0.2		f2	200	0.3	0.2
	f2	200	0.9	0.5	Patient_28	f1	200	0.3	0.1
Patient_14	f1	200	0.5	0.3			600	0.3	0.1
		600	0.4	0.1		f2	200	0.3	0.2
	f2	200	0.4	0.2			600	0.3	0.2
		600	0.3	0.2	Patient_29	f1	200	1.0	0.5
Patient_17	f1	200	0.4	0.2			600	1.2	0.5
		600	0.5	0.2		f2	200	0.4	0.1
	f2	600	0.2	0.1			600	0.3	0.1
Patient_18	f1	200	0.7	0.3	Patient_30	f1	200	0.6	0.2
	f2	200	0.5	0.3			600	0.4	0.2
		600	0.5	0.3		f2	200	0.3	0.1
Patient_20	f1	200	1.0	0.3			600	0.2	0.2
		600	2.1	0.9					
	f2	200	0.4	0.2					

The analysis of tumor motion between the adaptive protocols ADAPT_200 and ADAPT_600 demonstrated varying levels of motion. For the fast adapt scan (ADAPT_200), 80%, 13.3%, and 6.7% of scans had a difference in tumor trajectory between ADAPT_200 and the conventional scan of less than 1 mm, between 1 mm and 2 mm, and greater than 2 mm, respectively.

In contrast, for ADAPT_600, 76.9%, 15.4%, and 7.7% of scans had a difference in tumor trajectory between ADAPT_600 and the conventional scan of less than 1 mm, between 1  and 2 mm, and greater than 2 mm, respectively.

Patient 1 in fraction 1 shows the highest average distances of 2.4  and 2.3 mm for scan types ADAPT_200 and ADAPT_600, respectively, with corresponding standard deviations of 0.7  and 0.6 mm, respectively. In contrast, Patient 28 shows the lowest average distances of 0.3 mm for both adaptive scans for fraction 1 and average distances of 0.3 mm for fraction 2 for both adaptive scans with standard deviations of 0.2 , 0.1 , 0.2 , and 0.2 mm, respectively.

Comparing fractions 1 and 2, some patients exhibit differences in average distances and variability. For example, Patient 29 shows low average distances in fraction 2 (0.4 mm for ADAPT_200 and 0.3 mm for ADAPT_600) compared to fraction 1 (1.0  and 1.2 mm, respectively). This variation highlights the dynamic nature of tumor motion, which can vary not only between patients but also across different treatment fractions for the same patient. This observation aligns with findings in prior studies that have reported day‐to‐day variability in tumor motion due to factors such as respiratory patterns, patient setup, or anatomical changes during the treatment course.^21^, ^22^ Such variations underscore the importance of adaptive imaging protocols like ADAPT_200 and ADAPT_600, which aim to capture these changes with high precision. By accounting for these daily fluctuations, these protocols can enhance motion management strategies and improve overall treatment accuracy. Further exploration of this variability could help optimize imaging and treatment approaches, ensuring they are robust to day‐to‐day changes.

There are several patients, like patient 28, who exhibit consistent tumor motion across fractions and scan types, with average distances remaining low and stable (0.3 mm) for ADAPT_200 and ADAPT_600 in both fractions.

There was a small impact of the scan type on the average distances and their variability, and, as one might expect, there is a slightly higher variability for the lower projection count scan (ADAPT_200). For some patients, the ADAPT_600 scan type (high‐quality adaptive) shows lower average distances and standard deviations compared to the ADAPT_200 (fast scan).

The table highlights each phase's mean distances and standard deviations, providing a comprehensive overview of tumor motion variability between scan types.

**FIGURE 3:**
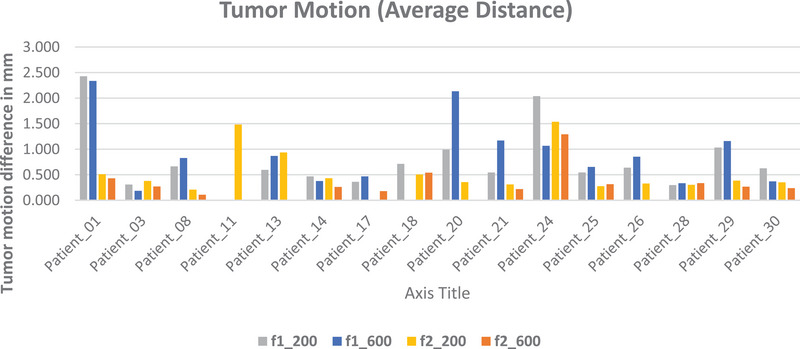
Comparison of average distance for scan types ADAPT_ 200 and ADAPT_ 600 in fraction 1 and fraction 2.

Figure 3 shows the average difference between tumor motion for each patient across two different protocols (ADAPT_200 and ADAPT_600) and two fractions (f1 and f2). The average distances are represented as f1_200, which represents fraction one under ADAPT_200 (grey bars), f1_600 represents fraction one under ADAPT_600 (blue bars), f2_200 represents fraction two under ADAPT_200 (yellow bars), and f2_600 represents fraction two under ADAPT_600 (orange bars). The graph showed that most patients experienced lower average tumor motion differences between the adaptive and conventional scans in the second fraction (f2) compared to the first fraction (f1) for both protocols. This could be because the patient has settled down a bit after the stress of the first fraction. For example, Patient_01 had a reduction in tumor motion from f1_200 to f2_200 and from f1_600 to f2_600. In 68.8% of patients, the ADAPT_200 protocol demonstrated a consistent reduction in average differences from fraction 1 to fraction 2. The ADAPT_600 protocol showed a varied response, with some patients experiencing decreased motion in the second fraction.

### 3.2 Qualitative analysis

**FIGURE 4:**
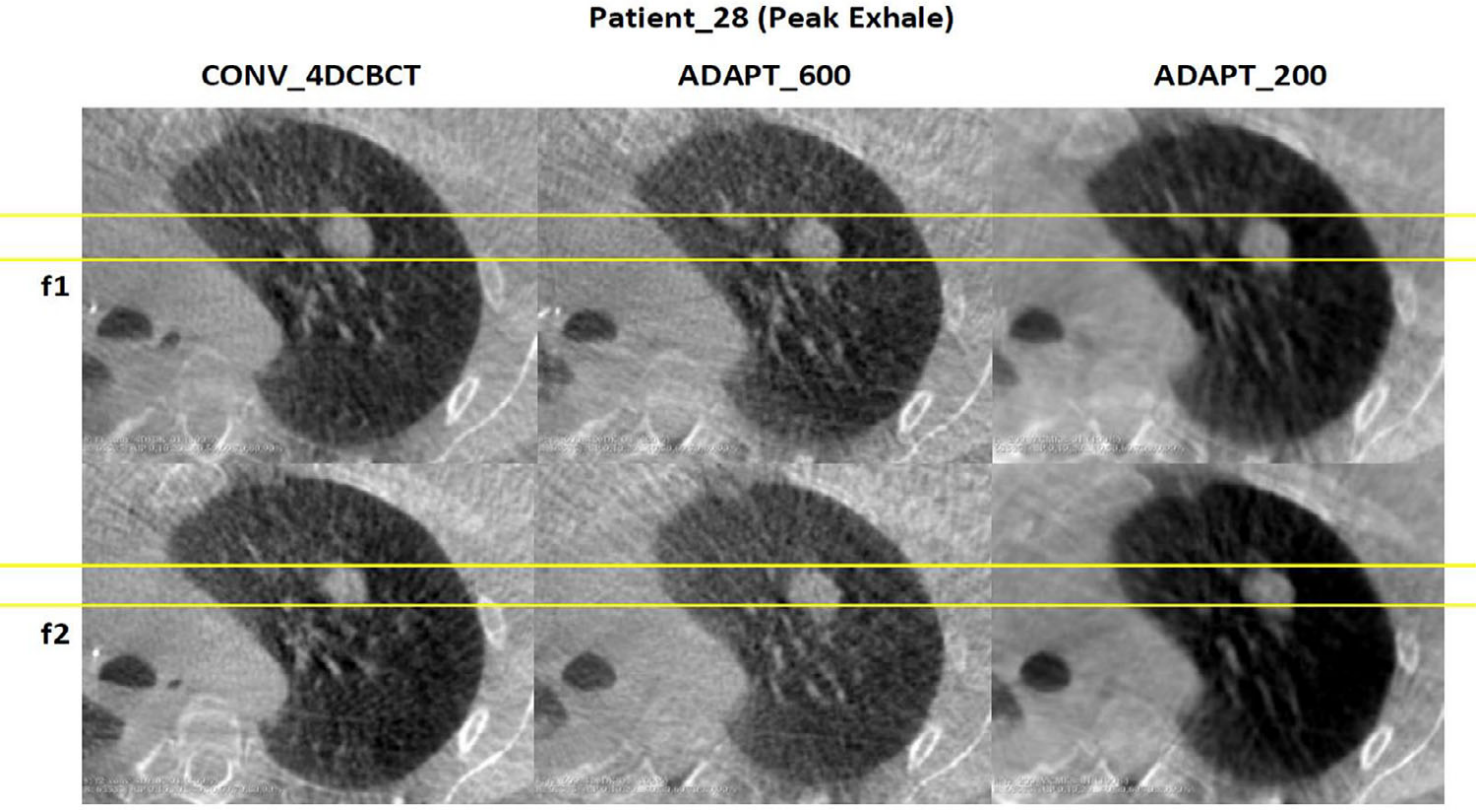
Visual assessment of the tumor for patient_28, which had the smallest average distance between the conventional scan and the adaptive scans.

**FIGURE 5:**
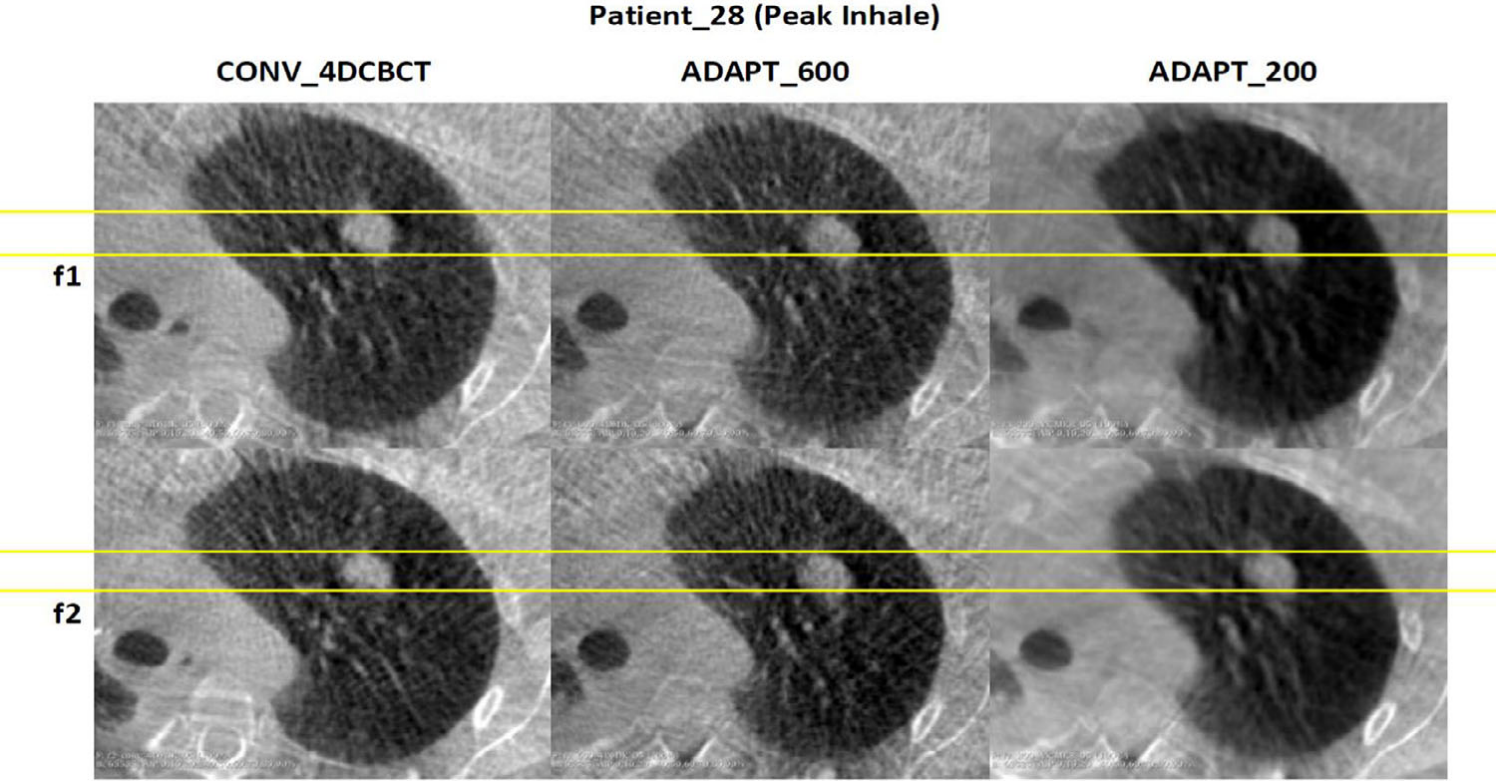
Visual assessment of the tumor for patients_28 with the smallest and almost constant average distance across different scan types and different fractions, during peak inhale across different imaging methods.

Figures 4 and 5 demonstrate the visual assessment of tumor motion for patient 28, who had the smallest mean difference across the patient cohort. The comparison is made between conventional 4DCBCT, ADAPT_600, and ADAPT_200 reconstructions during peak inhale and peak exhale phases. The comparison shows relatively minor differences in the three scans between peak inhale and peak exhale.

For ADAPT_600, there is a small difference in the scans on the posterior side, suggesting that variations in patient breathing or data acquisition methods during peak inhale and peak exhale can lead to slight but observable differences in tumor shape and motion. This is consistent with findings in the literature, where breathing variability during radiotherapy sessions has been shown to affect tumor motion patterns. For example, studies have demonstrated that reducing within‐patient breathing variability through techniques such as mechanical ventilation can improve consistency in radiotherapy delivery.^23^ Real‐time intrafraction motion monitoring has highlighted how breathing variations can impact tumor motion despite standardized imaging protocols.^24^ This is consistent in the literature, where variations in breathing and breathing regularity have been observed to impact 4DCBCT imaging of the tumor. This dynamic nature of breathing underscores the importance of adaptive scanning protocols like ADAPT_600, which are designed to account for these fluctuations while optimizing imaging accuracy and efficiency. Additionally, it is worth considering that alignment issues, such as slight variations in anatomical positioning even after rigid registration, could also contribute to the observed discrepancies.^7^, ^24^, ^25^

**FIGURE 6:**
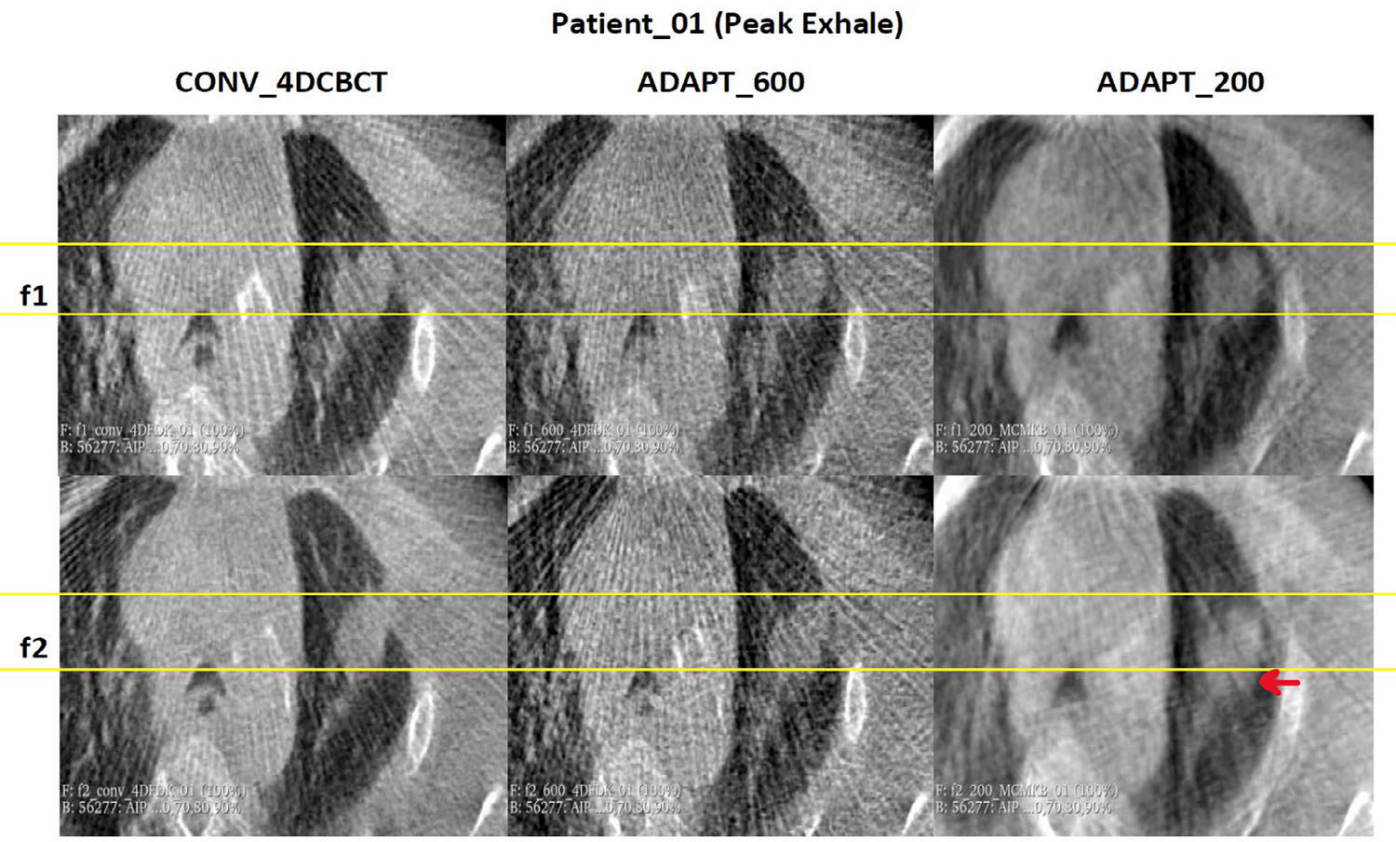
Visual assessment of the tumor for patient_01 with the largest average distance during peak exhale across different imaging methods.

**FIGURE 7:**
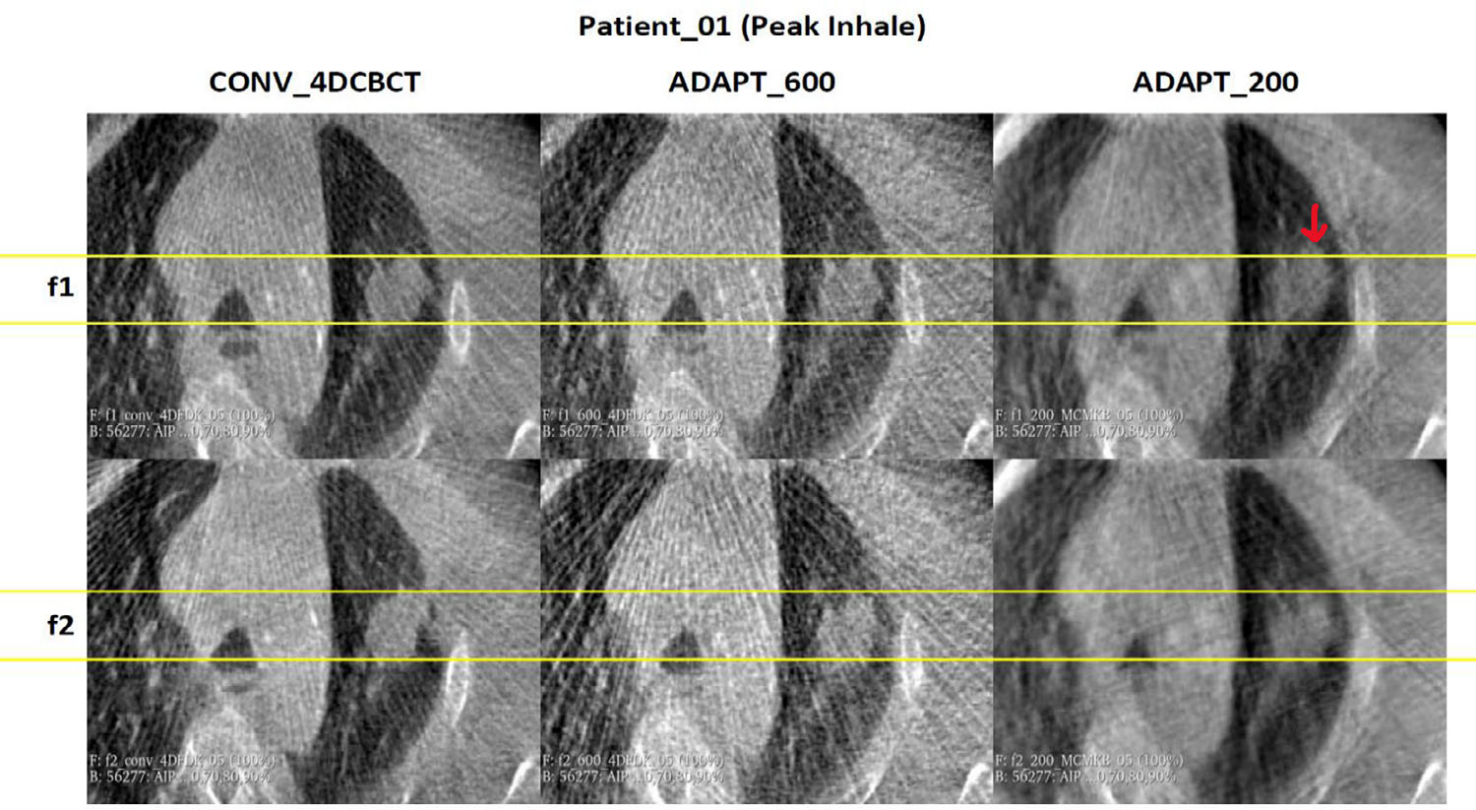
Visual assessment of the tumor for patient_01 with the largest average distance during peak inhale phases across different imaging methods.

Figures 6 and 7 show images for the patient with the largest average distance compared to the conventional scan. Table 1 indicates that for this patient, the average difference is above 2 mm for fraction 1, but for fraction 2, the average differences are much smaller. We can observe notable regions where there is a difference between the different scans (red arrows), which is made even more challenging for this patient as the tumor boundaries are harder to visualize. However, it appears that the tumor location is slightly different for this patient in the ADAPT_200 scan than for the other scans, and this may reflect the way that the patient was breathing during the scan which is evidenced by the location of the rib near the tumor that is in a different position across the scans. For patients with tumors that are harder to visualize, a higher projection count scan (ADAPT_600) might be preferred over the other scan types. An additional factor is that the breathing pattern can change between scans, with the ADAPT_200 scan acquired across fewer breathing cycles (20 breathing cycles) compared to ADAPT_600 and conventional (approximately 60 breathing cycles).

**FIGURE 8:**
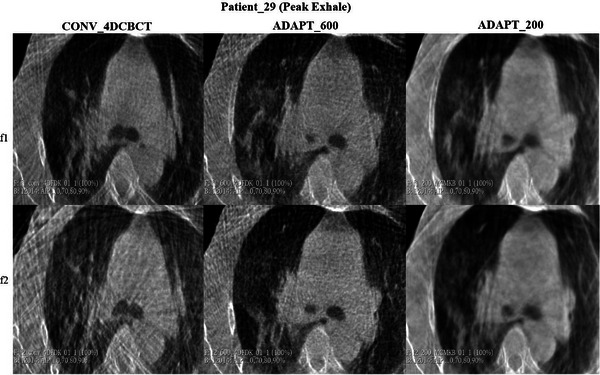
Visual assessment of tumor motion for patient_29 during peak exhale phase. The comparison includes conventional 4DCBCT, ADAPT_600, and ADAPT_200 scans across fractions 1 (f1) and 2 (f2).

For patient 29 (see Figure 8), the conventional scan had substantial streaking artifacts around the tumor, with the adaptive scans providing a clearer visualization of the tumor. Adaptive imaging ensures that projections are evenly spaced around the 200‐degree gantry rotation, which reduces streaking artifacts, especially for irregularly breathing patients.

**FIGURE 9:**
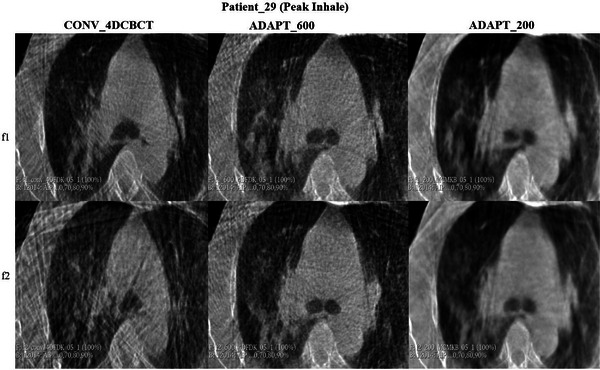
Visual assessment of tumor motion for patient_29 during peak inhale phase. The comparison includes conventional 4DCBCT, ADAPT_600, and ADAPT_200 scans across fractions 1 (f1) and 2 (f2).

Patient 29 in Figures 8 and 9 presents a challenging case in terms of image quality, characterized by integration of the tumor with soft tissue and noticeable streak artifacts in the conventional 4DCBCT scan, especially in fraction 2. These findings are consistent with other studies, where similar challenges were observed in visualizing tumor motion due to soft tissue integration and imaging artifacts.^26^, ^27^

## 4 DISCUSSION

This study analyzed tumor motion accuracy between adaptive scanning protocols, ADAPT_200 and ADAPT_600, and conventional 4DCBCT. The comparison of tumor motion trajectories between the ADAPT_200 and ADAPT_600 protocols highlights the effectiveness of both adaptive scanning protocols in capturing tumor motion. A large majority of the tumor motion for both protocols fell within the < 1 mm range, with 80% of the ADAPT_200 fractions and 76.9% of the ADAPT_600 fractions showing average differences to the conventional scan of less than 1 mm. Average differences between the 1  and 2 mm ranges showed that ADAPT_200 recorded 13.3% of fractions within this range, while ADAPT_600 exhibited 15.4%. This slight variation in percentage may be attributed to patient‐specific factors, such as differences in respiratory patterns during scanning. However, both protocols displayed a minimal percentage of cases where motion exceeded 2 mm, 6.7% for ADAPT_200 and 7.7% for ADAPT_600.

Overall, these findings support the use of adaptive protocols in routine clinical practice. The consistency in the results between ADAPT_200 and ADAPT_600 further enforces the potential benefits of adaptive imaging.

This study demonstrates that an ADAPT_200 scan accurately represents the tumor trajectory with a reduction of 75% of the scan time and 85% reduction in the total number of projections. The shorter scan times are expected to reduce patient discomfort and improve patient throughput without reducing image quality. The reduced number of projections still maintains a high level of accuracy in capturing the tumor's motion, which is critical for effective radiotherapy planning and delivery. The reduced scan times can cut almost 3 min off a treatment slot, which increases patient throughput but also reduces patient discomfort with shorter treatment slots.

The longer, high‐quality adaptive 4D CBCT scan, with 600 projections, results in a 55% reduction in imaging dose without substantially reducing image quality. This is particularly important in reducing radiation exposure to patients while still providing the necessary imaging detail for accurate tumor localization and motion tracking. The balance between image quality and radiation dose is crucial in clinical practice, and our study supports the use of high‐quality adaptive scans as a viable option, especially in cases where the tumor is harder to visualize or for irregular breathing patients where conventional 4DCBCT can suffer from streaking artifacts.

One of the main concerns of adaptive 4DCBCT is the use of motion‐compensated reconstruction methods, which have yet to find their way into routine clinical practice. Our results demonstrate comparable tumor trajectories using motion‐compensated and FDK reconstruction methods, indicating the feasibility of this approach in a patient cohort. This finding is as it supports the adoption of advanced reconstruction techniques that can potentially enhance image quality and accuracy without necessitating extensive changes to current clinical workflows.

The FDK algorithm achieves optimal performance when the projections are evenly distributed around the subject.^28^ Furthermore, it is essential to consider the natural variability in patient motion between scans and treatments. Previous studies^22^ have shown that both four‐dimensional computed tomography (4DCT) and four‐dimensional cone beam computed tomography (4DCBCT) can underpredict lung target motion during radiotherapy. Our observed changes in tumor motion fall within the bounds of these reported variations, suggesting that the average differences in motion we observed might be attributed to patient variability rather than the scanning technique.

In the case of patient_29, the worst‐case scenario in terms of image quality, the tumor was closely integrated with soft tissue, and the presence of streak artifacts, especially in conventional 4DCBCT scans. Despite these challenges, ADAPT_600 and ADAPT_200 scans provided consistent and reliable images for analysis with a reduced rate of streaking.

Step 2.5.2 of the workflow adjusts the ROI to remove bony anatomy from entering and exiting the ROI during breathing, which would influence the COM computation. Other soft tissue can still move in and out of the ROI, which could also impact the COM computation, but on visual inspection, these regions are small relative to the tumor volume, and therefore, we expect the COM to accurately reflect tumor motion for the majority of patients.

Tumour motion analysis in this study was not possible when there was bony anatomy in or near the cropped region. To further expand this study, an approach that can handle bony anatomy moving in and out of the cropped region would need to be developed.

In this study, tumor contouring was not performed for several reasons. First, contouring tumors on 4DCBCT images involves a workload and introduces interobserver variability, which can affect the reliability of the results. Additionally, the current lack of a universally accepted and reliable AI solution for automated tumor contouring adds to the complexity and potential inaccuracy of this approach. Furthermore, given the generally lower image quality of 4DCBCT compared to other imaging modalities, contouring on these scans is often not undertaken as the resulting contours may not be sufficiently accurate for clinical decision‐making. Instead, the focus was on evaluating the effectiveness of the ADAPT_200 and ADAPT_600 techniques in measuring tumor motion and optimizing imaging protocols, which provided valuable insights without the need for contouring.

The comparison of ADAPT_200 and ADAPT_600 scans with conventional 4DCBCT reveals that adaptive scanning techniques offer a reliable measurement of tumor motion throughout the respiratory cycle, including peak inhale and exhale phases. The results suggest that adaptive scans can provide improved accuracy in capturing tumor motion, potentially enhancing treatment precision in clinical practice. This is the first study to examine tumor motion using the ADAPT_200 and ADAPT_600 protocols in a cohort of lung cancer patients, marking a key step toward clinical translation of these adaptive imaging methods. The primary clinical use of 4DCBCT in radiotherapy is to assess intrafraction tumor motion and verify tumor positioning, particularly in the thoracic and abdominal regions. Although ADAPT_200 demonstrates slightly reduced image quality compared to ADAPT_600, the motion information remains sufficiently accurate for clinical evaluation. The trade‐off between reduced acquisition time, lower dose, and minor degradation in image quality supports the continued usability of ADAPT_200 for motion assessment in routine treatment workflows.

In this study, the diversity in body weights, ranging from 52.2  to 95.1 kg, and the various tumor stages (T0 to T4 and TX) indicate that the adaptive 4DCBCT techniques were tested across a reasonable spectrum of patient profiles. This variation is crucial for understanding the performance of adaptive 4DCBCT in different clinical scenarios. The predominance of patients in the early stages of cancer (T1 and T2) underscores the potential of adaptive 4DCBCT for early detection and precise monitoring of tumor motion. The ability to accurately capture tumor motion in patients with varying demographics and tumor stages can lead to more personalized and effective radiotherapy treatments.

Despite employing advanced reconstruction techniques in ADAPT_600 and ADAPT_200 scans, our study faced challenges in regard to the generally low quality of 4DCBCT imaging. While 4DCBCT imaging generally provides adequate image quality for patient positioning and motion assessment, enhancing image clarity and precision could further boost the clinical team's confidence in tumor motion measurements. This underscores the need for continued refinement of imaging techniques, especially in complex clinical scenarios.

In this study, baseline shifts were accounted for through rigid registration to minimize their influence on the observed COM differences. However, variations in respiratory patterns remain a factor that could impact the tumor motion observed in the post‐treatment scans compared to the pre‐treatment scans. While differences in acquisition and reconstruction methods may contribute to these variations, patient‐specific breathing irregularities could also play a role. To better distinguish imaging‐related discrepancies from those caused by respiratory variability, further studies are required to analyze the extent of breathing irregularity and its influence on motion measurements.

This is the first study to analyze tumor motion using ADAPT_200 and ADAPT_600 in a lung cancer patient cohort, which is an important and necessary step toward clinical translation. Various other reconstruction and acquisition protocols have been investigated in the literature, such as iterative reconstruction techniques^29^, ^30^ and slower gantry rotations.^31^ In the adapted clinical trial, we selected adaptive imaging to reduce the imaging dose compared to slow gantry rotation acquisitions and the MCMKB image reconstruction^15^ pipeline to overcome the long reconstruction times (hours) of iterative methods.

The findings of this study have implications for radiation oncology practice. ADAPT_200 and ADAPT_600 offer notable advantages over conventional 4DCBCT, including reduced scan times and decreased patient radiation exposure. These techniques ensure accurate tumor trajectory measurements, facilitating precise treatment targeting and better protection of healthy tissues. Our results support the integration of advanced reconstruction methods, such as motion‐compensated techniques, into clinical workflows, demonstrating their potential to enhance treatment precision without causing workflow disruptions.

## 5 CONCLUSION

The ADAPT‐200 scan demonstrates potential in accurately measuring tumor trajectories while offering substantial benefits in both scan time and imaging dose. Specifically, our study found that the ADAPT_200 scan can achieve a reduction in scan time by up to 75% and a decrease in imaging dose by 85% compared to conventional 4DCBCT scans. This reduction in scan time not only reduces patient discomfort but also enhances clinical efficiency by allowing more patients to be scanned within the same timeframe. Moreover, the lower imaging dose reduces the radiation exposure to patients, thereby decreasing the risk of radiation‐induced side effects. In this study, these improvements did not come at the cost of accuracy. Given these advantages, the ADAPT scans present a promising alternative to conventional 4DCBCT, with the potential to be integrated into routine clinical practice to improve patient care and treatment outcomes.

## AUTHOR CONTRIBUTIONS

Sadia Sana conceptualized the study, designed the methodology, conducted data analysis, compiled the results, and drafted the manuscript.

Owen Dillon contributed to data interpretation, conducted clinical trials, provided technical insights, validated the results, and assisted in manuscript revision, ensuring the accuracy of the findings.

Ricky T. O'Brien conceptualized the study, conducted clinical trials, supervised the research, provided critical guidance on study design and methodology, contributed to result validation, and played a key role in refining the manuscript.

All authors read and approved the final manuscript.

## CONFLICT OF INTEREST STATEMENT

Authors O'Brien and Dillon are inventors of a licensed patent or IP related to this technology.
